# Combining noninvasive electrocardiographic imaging mapping method with fluoroscopic integration module enables the idiopathic ventricular fibrillation ablation triggered by rare premature ventricular contraction

**DOI:** 10.1016/j.hrcr.2024.12.004

**Published:** 2024-12-11

**Authors:** Reşit Yiğit Yılancıoğlu, Oğuzhan Ekrem Turan, Mahmut Mustafa Barış, Emin Evren Özcan

**Affiliations:** Faculty of Medicine, Department of Cardiology, Heart Rhythm Management Center, Dokuz Eylul University, Izmir, Turkey

**Keywords:** Premature ventricular complex, ECG imaging, Fluoroscopy integration module, Radiofrequency catheter ablation, Idiopathic ventricular fibrillation

**Key Teaching Points**

• This case report highlights the effectiveness of integrating noninvasive electrocardiographic imaging with invasive fluoroscopy-based mapping to precisely localize and ablate rare premature ventricular contractions in patients with idiopathic ventricular fibrillation.
• Innovative solutions, such as the "tattoo method" for stable electrocardiographic electrode positioning and advanced mapping technologies, are crucial for managing complex arrhythmias originating from difficult anatomic locations, such as the right ventricular moderator band.
• Patients with idiopathic ventricular fibrillation often present with a very low burden of premature ventricular contractions, making it challenging to map and localize the arrhythmogenic focus. Traditional invasive mapping techniques can be problematic because of the risk of inducing ventricular fibrillation during catheter manipulation.

## Introduction

Idiopathic ventricular fibrillation (IVF) is a primary cause of sudden cardiac death in young patients, especially in those without any structural or electrical heart abnormalities.^1^ Premature ventricular contractions (PVCs) inducing IVF have been reported to be particularly related to short-coupled PVCs (coupling interval ≤350 milliseconds), which mostly originate from Purkinje systems.^2^, ^3^, ^4^ Invasive mapping of originating PVCs can be difficult due to VF induction by catheter manipulation or pace-map and anatomic difficulties. We aimed to present novel invasive management of a patient with IVF with a very low PVC burden (<1%), guided by combining the fluoroscopy integration module and a noninvasive electrocardiographic vector imaging (ECGvi) mapping technique.

## Case report

**Figure 1:**
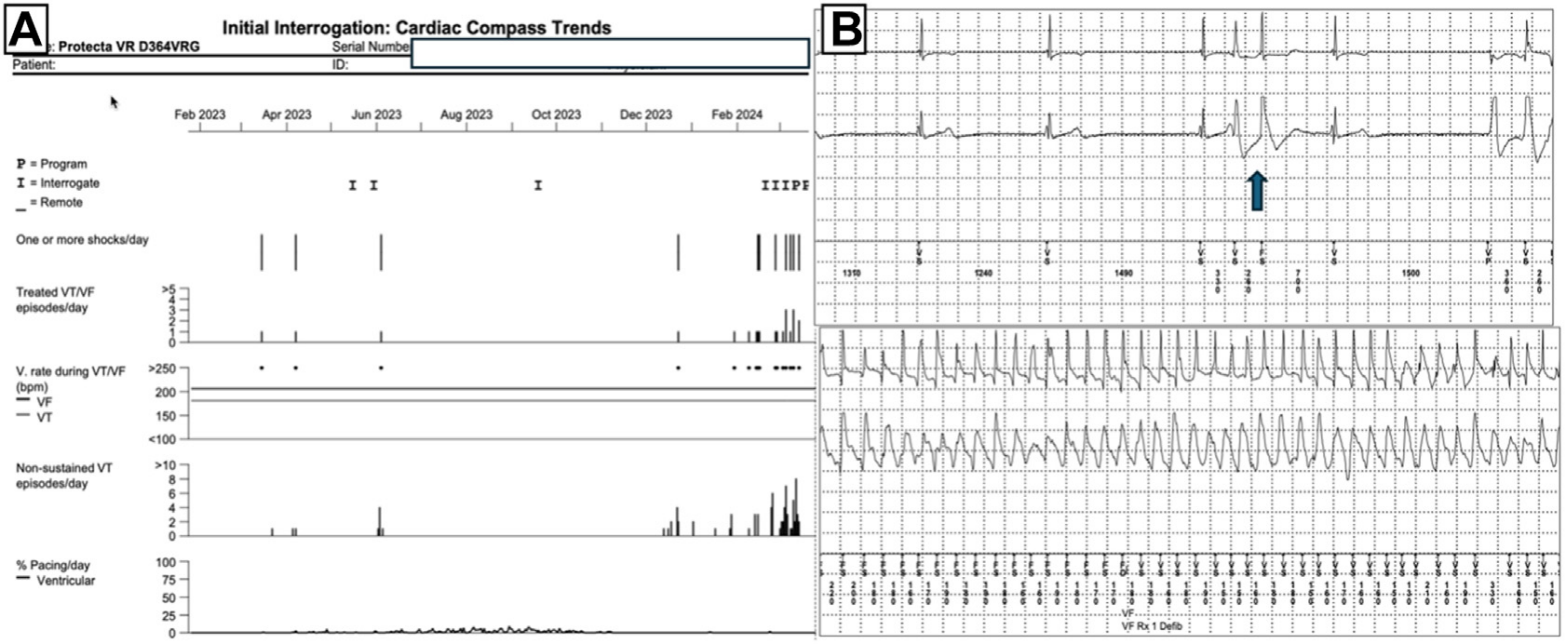
Implantable cardioverter-defibrillator (ICD) interpretation. **A:** Frequency of direct current shocks in the compass section of the ICD. **B:** A shock episode that is initiated with a single premature ventricular contraction (*blue arrow*) and continues polymorphic ventricular tachycardia/ventricular fibrillation (VT/VF) and shock delivered.

**Figure 2:**
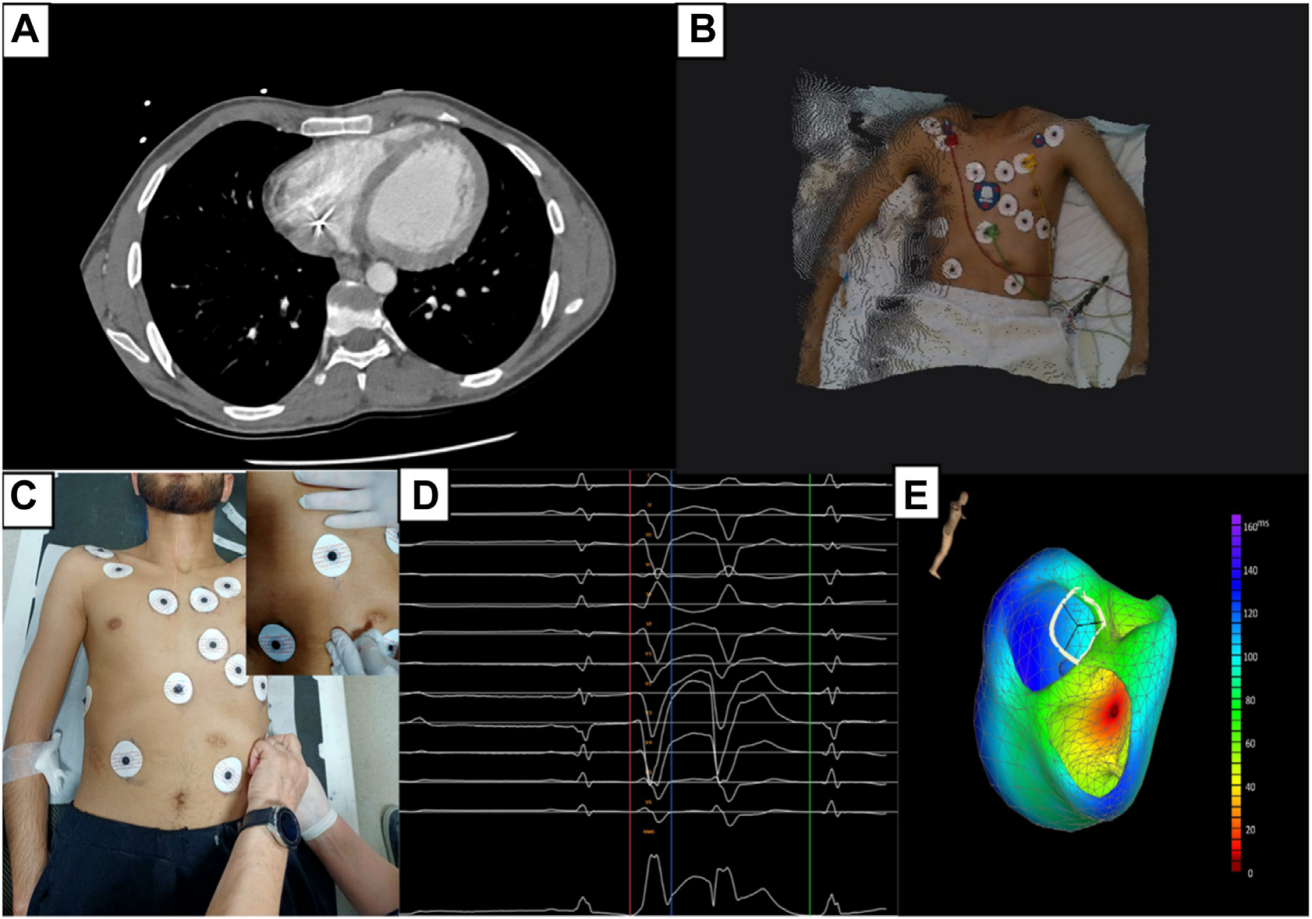
How to collect the premature ventricular contraction (PVC) template in very rare PVC burden with idiopathic ventricular fibrillation for noninvasive activation mapping precision tool. **A:** Cardiac computed tomography (CT) performed. **B:** Body 3-dimensional (3D) photograph was obtained via VIVO device with landmark patches. **C:** Tattoage, which keeps the electrode in the correct position out of hospital, was performed to increase accuracy of 3D body photography. Tattoage was used to mark the electrodes at the 6 o’clock position by needle imprint method. **D:** PVC collected and transferred to VIVO module.

**Figure 3:**
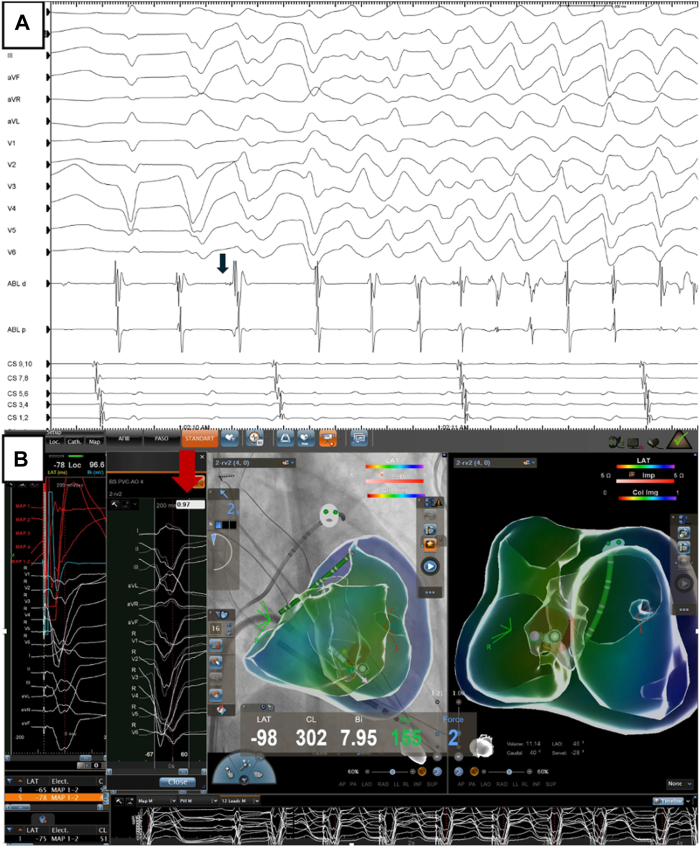
**A:** Ventricular fibrillation episode induced during mapping by catheter contact. *Blue arrow:* Purkinje potential. **B:** Catheter-associated premature ventricular contractions (PVCs) have a high match score with clinical PVC. *Red arrow:* PVC PASO score.

A 22-year-old male patient presented to the emergency department with recurrent implantable cardioverter-defibrillator (ICD) shock and syncope that occurred more than once per month. The patient’s clinical history included secondary prevention ICD implantation due to aborted sudden cardiac death 10 years earlier. The patient did not have any structural cardiac disease. Previously, genetic tests for hereditary arrhythmias and cardiomyopathy were unremarkable. Baseline 12-lead electrocardiogram (ECG) showed sinus rhythm and no relevant abnormalities. Interrogation of the ICD showed that patients with all ICD therapies were treated for VF episodes that were caused by PVCs (Figure 1A and 1B). Echocardiography revealed no structural abnormalities or prominent moderator band in the right ventricular (RV) chamber. During long-term follow-up in the intensive care unit, no PVCs were observed during continuous rhythm monitoring. PVCs were not induced with isoprenaline infusion or conscious sedation. The ECGvi method was planned for use as a guiding imaging method because of the rarity of PVC. Preparation for ECGvi was planned. Cardiac computed tomography was performed according to the ECGvi algorithm as View into Ventricular Onset (VIVO, Catheter Precision, Ledgewood, NJ) protocol (Figure 2A). The computed tomography image was imported into VIVO software. A 3-dimensional (3D) topographical photograph of the patient's chest was obtained, and 3 reference points of localization positioning patches were placed on the patient's chest (Figure 2B). ECG morphology could not be collected for the template because PVCs were not detected during the index hospitalization. Therefore, we aimed to protect the electrodes and reference points using a tattoo. The needle imprinting tattoage method was used to ensure stable ECG electrode positions in the patient, with radiation oncology patient preparation unit support (Figure 2C). Rare PVCs occurred 10 days after discharge. ECG morphology was collected with the CARTO System (Biosense Webster, Irvine, CA) and electrophysiology recording system CardioLab (GE Healthcare USA, Chicago, IL), and uploaded to the VIVO system. The VIVO system was used to create an ECGvi map of endocardial and epicardial shell forms (Figure 2D and 2E). The file was saved and transferred to the CARTO system as a Visualization Toolkit file format via a USB drive. The case file was imported into the CARTO System by selecting an associated study from the CARTO Merge Module. Registration was carried out within the CARTOUNIVU System (Biosense Webster) via 3D visual alignment. Right ventriculography was performed to determine the left and right anterior oblique angles for integration with the CARTOUNIVU module. The CARTOUNIVU module and VIVO map had integrated each other (Supplemental Video 1), Thus, the electroanatomic borders were more precise. A steerable long sheath was used to increase the stability. A 3.5-mm irrigated tip ablation catheter (SmartTouch SF, Biosense Webster) was used to measure the contact force. An activation map was not achievable interprocedurally because no PVCs were detected during the procedure. The patient was agitated and deep sedation was required. We attempted to create a pace map with an output of 4–5 mA and a pulse width of 1 millisecond, but VF occurred several times during the mapping. During mapping near the moderator band, this electrogram showed the Purkinje potential and then the rhythm suddenly induced VF (Figure 3A and 3B). We could not create a reliable pace map. The PVC was localized to the RV moderator band’s septal exit and co-localized to the predicted PVC on the registered VIVO map), and 5 ablation lesion sets were delivered during the procedure, which lasted 120 minutes (Supplemental Figure 1). The patient did not experience any ICD shock or episodes during the 3-month follow-up period, without antiarrhythmic drugs.

## Discussion

A case of IVF induced by PVCs presents several challenges. In our case, most of the early PVCs degenerated into the VF and PVCs were too rare to be mapped. Catheter contacts easily induced VF. Therefore, PVCs had to be mapped with a noninvasive mapping technique.

IVF, which occurs in the absence of underlying structural heart disease or ECG abnormalities and represents a subgroup of patients who survive out-of-hospital sudden cardiac death. Despite therapy with an ICD, arrhythmias can recur and even lead to electric storms. In our case, short-coupled PVC episodes may have caused VF. This can lead to a vicious cycle. The 12-lead ECG morphology of the PVC was suggestive of an RV inflow PVC origin with left bundle branch block morphology and a very late transition superior axis. Noninvasive preprocedural 3D mapping with VIVO confirmed this location by considering the individual 3D anatomy of the patient. Although several beats were sufficient for noninvasive mapping, no PVCs were obtained in our patient, despite induction methods, such as isoprenaline, atropine, or sedation. Because ECG electrodes serve as anatomic markers, it is important to ensure that they are correctly localized. Lesina and colleagues^5^ reported that noninvasive activation mapping with VIVO is sensitive to changes in anatomic model and time marker placement, but less sensitive to changes in body position. A needle imprint tattoo was used to mark the electrodes at the 6 o’clock position to ensure consistent placement. Permanent ink tattooing remains the most widely used and cost-effective method of skin marking, especially for radiotherapy.^6^ We used tattoos to keep the electrode in the correct position outside the hospital because of the increased accuracy of ECGvi. After 10 days, when the patient’s PVCs appeared, the same computed tomography and topography could be used (Figure 3).

The recommended strategy for IVF ablation is to target the site of earliest ventricular activation during spontaneous PVCs. In patients without clinical PVCs, ablation can target local Purkinje potentials or the site with the best-matched morphology by pace mapping. During ablation, exacerbation of arrhythmias, such as VF, is common before premature beats are eradicated.^7^ In the case of VF with catheter manipulation, mapping and ablation may be complicated. As in our case, these VF triggers may be related to potentials originating from the Purkinje fibers. These triggers are commonly located in the anterior wall of the right ventricle, papillary muscles, or the RV moderator band in literature.^8^ The moderator band in the right ventricle extends from the septum to the free wall and is a muscular structure that encompasses the RV Purkinje fibers.^9^ The challenge of catheter contact and stability, which results in insufficient energy delivery to this thick intracavitary structure, is a potential mechanism for recurrence or failure.^10^ We could not effectively use pace-map or activation mapping for PVC mapping due to VF induction. The use of a combination of fluoroscopy integration module and noninvasive 3D mapping enabled the understanding of patient anatomy and consequently guided the operator to the appropriate location to achieve complete elimination of the PVC. Therefore, we were not required to create a 3D anatomic map using the catheter. In addition, Sadek and colleagues^11^ reported that intracardiac echocardiography may be a good tool for catheter ablation in the moderator band or inflow location; however, it was not available.

Concepts for the use of the ECG imaging tool are available in the literature.^12^ The combined use of noninvasive mapping with the ECG imaging tool is increasing. A recently published case report demonstrated that successful ablation was achieved with the integration of the VIVO module and remote magnetic imaging.^13^ Our case highlights the role of noninvasive ECG imaging mapping (VIVO System) with a fluoroscopy integration module (CARTOUNIVU Module) in innovative collaboration with the invasive management of recurrent malignant ventricular arrhythmias in young patients with IVF with hidden PVC triggers.

## Conclusion

Using advanced imaging techniques and innovative mapping technologies, we may achieve precise localization of arrhythmogenic substrates and help identify targeted ablation therapy, thereby mitigating the risk of recurrent arrhythmia-related complications and improving patient outcomes.

## Disclosures

The authors have no conflicts of interest to disclose.
