# Supplementary material for: Combining noninvasive electrocardiographic imaging mapping method with fluoroscopic integration module enables the idiopathic ventricular fibrillation ablation triggered by rare premature ventricular contraction
Source: HeartRhythm Case Rep. 2024 Dec 11;11(3):247–51. doi: 10.1016/j.hrcr.2024.12.004 (PMC11962992; doi:10.1016/j.hrcr.2024.12.004)
Supplement: Supplement Data [file mmc1.docx]

**Supplemental Data:**

**Supplemental figure 1:** Final lesion set with fast anatomical mapping (FAM) in non-invasive mapping. Red dots: Ablation dots. Red area predicted earliest point (PVCs exit) in non-invasive mapping (VIVO)


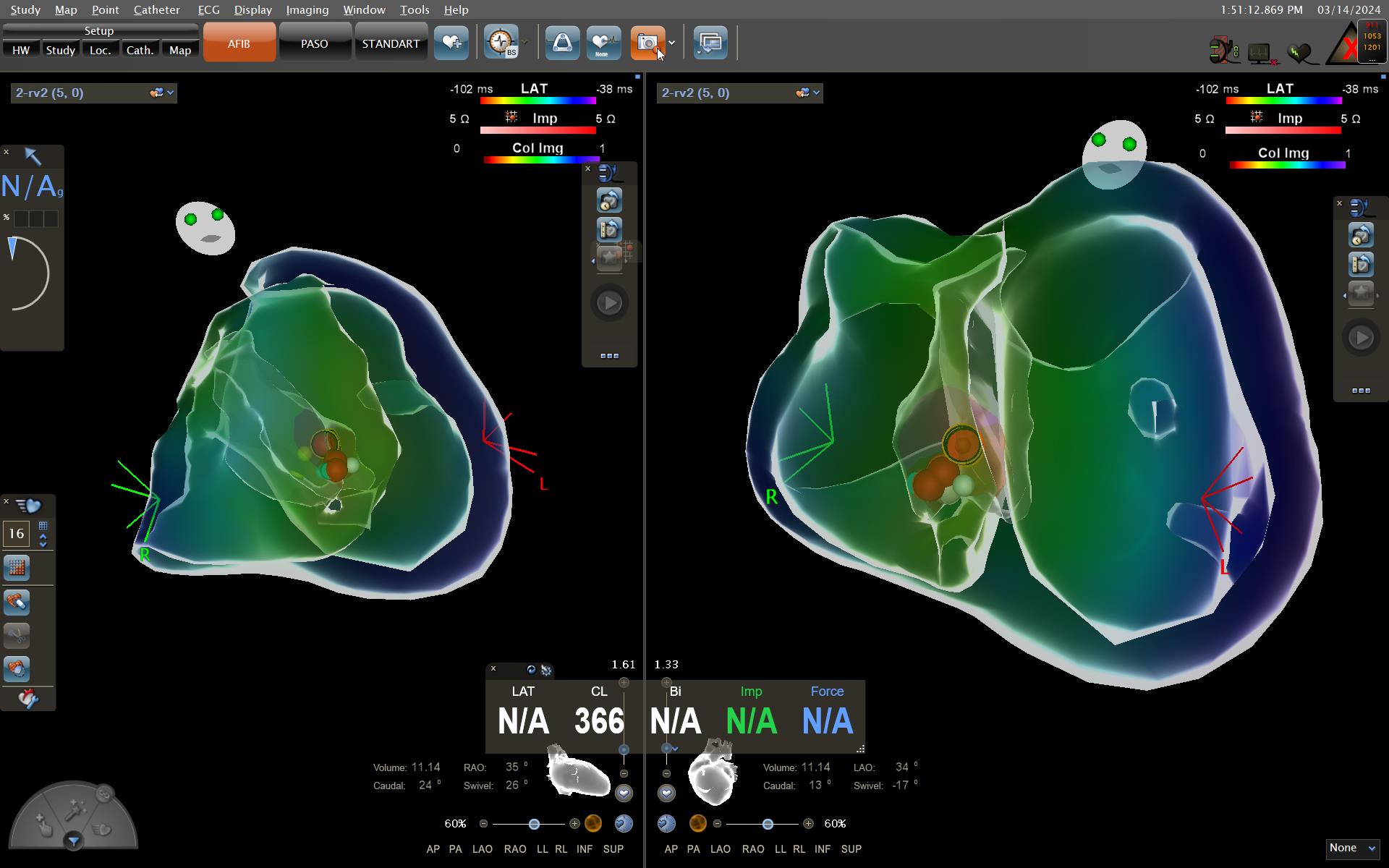


**Supplemental video 1: How to The Carto UniVU module and VIVO map was integrated**
